# Gut Microbiota and the Neuroendocrine System

**DOI:** 10.1007/s13311-017-0600-5

**Published:** 2018-01-27

**Authors:** Aitak Farzi, Esther E. Fröhlich, Peter Holzer

**Affiliations:** 1 Otto Loewi Research Center, Pharmacology Section, Medical University of Graz, Graz, Austria; 2 BioTechMed-Graz, Graz, Austria

**Keywords:** Antibiotics, Corticosterone, Germ-free mice, HPA axis, Probiotics, Stress

## Abstract

The microbial ecosystem that inhabits the gastrointestinal tract of all mammals—the gut microbiota—has been in a symbiotic relationship with its hosts over many millennia. Thanks to modern technology, the myriad of functions that are controlled or modulated by the gut microbiota are beginning to unfold. One of the systems that is emerging to closely interact with the gut microbiota is the body’s major neuroendocrine system that controls various body processes in response to stress, the hypothalamic–pituitary–adrenal (HPA) axis. This interaction is of pivotal importance; as various disorders of the microbiota–gut–brain axis are associated with dysregulation of the HPA axis. The present contribution describes the bidirectional communication between the gut microbiota and the HPA axis and delineates the potential underlying mechanisms. In this regard, it is important to note that the communication between the gut microbiota and the HPA axis is closely interrelated with other systems, such as the immune system, the intestinal barrier and blood–brain barrier, microbial metabolites, and gut hormones, as well as the sensory and autonomic nervous systems. These communication pathways will be exemplified through preclinical models of early life stress, beneficial roles of probiotics and prebiotics, evidence from germ-free mice, and antibiotic-induced modulation of the gut microbiota.

**Electronic supplementary material:**

The online version of this article (10.1007/s13311-017-0600-5) contains supplementary material, which is available to authorized users.

## Introduction

The neuroendocrine system is classically defined as an organized set of cells with neural determination, which produce hormones or neuropeptides [1, 2]. The hypothalamic–pituitary–adrenal (HPA) axis is considered to be the major neuroendocrine system regulating various body processes in response to psychological stressors and physical stressors, including infections, ensuring an adequate response to the stressor [3]. Corticotropin-releasing factor (CRF), the principle regulator of the HPA axis, is released from the paraventricular nucleus (PVN) of the hypothalamus in response to stress and induces the release of adrenocorticotropic hormone (ACTH) into the systemic circulation. ACTH subsequently induces the secretion of glucocorticoids (cortisol in humans and corticosterone in rodents) from the adrenal cortex [3]. The release of glucocorticoids, in turn, leads to a feedback inhibition through binding of glucocorticoids to glucocorticoid receptors (GR) localized in stress-responsive brain regions via transcriptional modulation [4].

Emerging evidence points to a bidirectional communication between the neuroendocrine system and gut microbiota, the complex community of microorganisms that resides in the mammalian intestinal tract. The establishment of the gut microbiota in early life has been increasingly recognized to influence several aspects of brain function and behavior, including neuroendocrine responses to stress [5, 6]. A potential link between the gut microbiota and neuroendocrine system is further suggested by disorders that are associated with disturbances in both systems, such as depression and irritable bowel syndrome (IBS). Increased activation of the HPA axis has been repeatedly observed in depressed patients, especially in the melancholic subtype [7]. While assessment of HPA axis activity in IBS has yielded inconsistent results, most studies support increased activity of the HPA axis in IBS [8]. Furthermore, early life stress, as well as chronic stressors, are risk factors for the development of depression and IBS [9–11]. While chronically elevated cortisol levels negatively affect brain function [11, 12], HPA axis activation is also able to affect the composition of the gut microbiota and increase gastrointestinal permeability [13, 14]. Dysbiosis of the gut microbiota and bacterial translocation have been proposed to contribute to the chronic low-grade inflammation that is observed in subsets of patients with IBS or depression [15, 16]. While not entirely consistent, several studies have reported an increased abundance of Firmicutes, whereas the abundance of Bacteroidetes was decreased in at least a subset of patients with IBS [17, 18]. Changes in microbiota composition are also reported in depressed patients, whereas no microbial signature for this condition has yet been defined [19–21]. A causal role of the microbiota in the development of both depression and IBS has further been demonstrated by fecal transplantation studies. Transplantation of microbiota of depressed patients to rodents is able to induce depression-like behavior [22]. Kelly et al. [19] observed anhedonia and anxiety in rats in response to transplantation of fecal microbiota from depressed patients. While microbiota transplantation induced indices of immune activation, stress-induced corticosterone levels were not affected [19]. While this result is rather surprising, measuring HPA axis parameters at a single time point could have yielded unreliable results [19]. Transplantation of fecal microbiota from patients with IBS with diarrhea (IBS-D) to germ-free (GF) mice with or without anxiety induced faster gastrointestinal transit, intestinal barrier dysfunction, innate immune activation, and anxiety-like behavior [23]. Immune activation in the recipient mouse colon was especially pronounced when the fecal microbiota derived from patients with IBS-D who also had anxiety-like behavior. These mice also displayed enhanced expression of colonic genes that are involved in GR pathway signaling [23]. Although the functional significance of the microbiota in the neuroendocrine disturbances of these multifactorial disorders has yet to be determined in detail, it is plausible that the changes in intestinal permeability and immune parameters could also contribute to the observed neuroendocrine abnormalities [24]. This contribution reviews largely preclinical studies that suggest communication between the gut microbiota, the neuroendocrine system, and the brain, which take place via multiple direct and indirect pathways including: 1) humoral pathways (via microbial metabolites, gut hormones) [25, 26]; 2) immune pathways (including proinflammatory cytokines) [27] and 3) neural pathways (via enteric nervous system, vagus nerve, and spinal afferents) [28].

## Early Life Events and the HPA Axis

Early life events are able to persistently program the HPA axis. While positive experiences such as maternal care and neonatal handling may lead to a dampened HPA response later in life [29, 30], exposure to stressful events can cause maladaptation of the HPA axis, providing a mechanistic basis for changes in stress susceptibility later in life [31]. Thus, early life stress typically results in increased HPA responsiveness to stress, which is due to increased CRF signaling and an impaired GR-mediated negative feedback [31]. Among other mechanisms, the effects of early life stress are brought about by increased corticosteroid exposure of the developing brain, leading to changes in GR expression [31, 32]. In addition, early life stress affects the function of various areas of the brain that are able to modulate the HPA axis, such as the amygdala, hippocampus, and prefrontal cortex [31]. While, in general, early life stress leads to hyperactivity of the amygdala [33], it inhibits synaptic plasticity and decreases the expression of NR_1_ and NR_2B_ subunits of the *N*-methyl-D-aspartate receptor (NMDA) receptor in the hippocampus [34], an area that exerts inhibitory effects on the HPA axis. Likewise, the prefrontal cortex is able to inhibit the activity of the HPA axis, with early life stress impairing the function of the prefrontal cortex through various mechanisms [31, 35]. Furthermore, noradrenergic neurons originating mainly from the nucleus of the solitary tract in the brainstem play a pivotal role in activating the HPA axis [36]. The nucleus of the solitary tract is of particular importance with regard to the gut–brain axis, as it receives visceral afferents from the vagus nerve and is activated by inflammatory challenges [37, 38].

Another hallmark of early life stress is an increase in the activity of the innate immune system that persists into adulthood, whereas the acquired immune system is rather impaired [39, 40]. The HPA axis is mechanistically involved in this response, as chronic activation of the HPA axis leads to a compensatory decrease of GR signaling through epigenetic changes [41], resulting in resistance of immune cells to the anti-inflammatory properties of cortisol [39, 42]. Emerging evidence also indicates a role of the gut microbiota and intestinal permeability in this process, as stress affects the composition of the gut microbiota [43] and weakens the intestinal mucosal barrier [14]. Stress-induced changes in gut microbiota composition could be induced by neuroendocrine hormones, such as norepinephrine (NE) and dopamine (DA), which have long been known to increase the growth of Gram-negative bacteria [44]. Increased intestinal permeability, in turn, has been proposed to induce an inflammatory response through translocation of bacterial constituents across the intestinal lumen [13]. Finally, inflammatory mediators, including proinflammatory cytokines and prostaglandins are potent activators of the HPA axis [45], adding to the complexity of microbiota-immune-neuroendocrine interactions.

## Gut Microbiota as the Source of Microbial Constituents that Activate the HPA Axis

While it is clear that inflammatory processes lead to activation of the HPA axis through the release of proinflammatory cytokines and prostaglandins, the mechanisms behind the contribution of the gut microbiota as a stimulator of the immune system are being extensively investigated. There are indices that activation of the HPA axis by the gut microbiota can occur as a result of increased permeability of the intestinal barrier and a microbiota-driven proinflammatory state [13]. Recently, it has been suggested that gut microbiota-derived peptidoglycan, the cell wall constituent of most bacteria, can translocate into the brain and activate specific pattern recognition receptors of the innate immune system and thereby affect brain development and behavior [46]. In addition, gut microbiota-derived peptidoglycan has been demonstrated to activate innate immunity by activating one of its receptors, the nucleotide-binding, oligomerization domain-containing protein-1 (Nod1) [47]. Apart from peptidoglycan, lipopolysaccharide (LPS), a constituent of the outer membrane of Gram-negative bacteria and activator of the Toll-like receptor (TLR) 4, has been proposed to cross the intestinal epithelial barrier in response to certain conditions such as stress or a high-fat diet (HFD) and thereby to lead to immune and HPA axis activation. The translocation and interaction of various bacterial constituents and their effects on the immune system and HPA axis could be of broad pathophysiological relevance. In this regard, synergistic interactions have been reported for LPS and Nod agonists, as well as LPS and the TLR2 agonist lipoteichoic acid [48, 49].

While bacterial constituents are able to acutely activate the immune system and HPA axis, exposure of newborn rodents to these factors is able to induce long-lasting effects. Thus, similar to stress, neonatal exposure to LPS leads to elevated ACTH and corticosterone responses to restraint stress and a decreased glucocorticoid feedback inhibition in adulthood, which is exemplified by an attenuated dexamethasone-induced suppression of ACTH responses to restraint stress [50]. Concordantly, neonatal LPS exposure decreases cerebral GR density, whereas CRF expression is increased [50]. In addition, neonatal exposure to LPS induces enhanced prostaglandin-mediated HPA axis reactivity to LPS in adulthood [51]. In addition to affecting the HPA axis, neonatal exposure to LPS is able to reprogram catecholaminergic neurons [52]. Thus, LPS-challenged neonates show marked increases in tyrosine hydroxylase activity and protein expression in adulthood, in particular in the locus coeruleus [52].

## Microbial Short-Chain Fatty Acids: Central Effects and Inducers of Gut Hormone Release

The ability of microbes to ferment indigestible carbohydrate fibers and convert them into short-chain fatty acids (SCFAs) is emerging to be of special importance in a variety of physiological and pathophysiological processes. Acetic acid, butyric acid, and propionic acid are the most widely studied SCFAs and have diverse functions within and outside the gastrointestinal tract. Locally, SCFAs are important energy sources for the gut microbiota itself and for intestinal epithelial cells. They enhance the integrity of the intestinal epithelium, increase mucus production, modulate gut motility, and exert anti-inflammatory effects, such as inactivation of nuclear factor kappa B and the promotion of regulatory T cells [53–55]. Furthermore, they induce the release of hormones and neuropeptides, such as glucagon-like peptide 1 (GLP-1) and peptide YY (PYY) from intestinal enteroendocrine cells [56]. On the one hand, these effects are mediated through activation of the G protein-coupled receptors (GPRs) GPR43, GPR41, and GPR109A, whereas, on the other hand, SCFAs are also epigenetic regulators, affecting gene expression by acting as inhibitors of histone deacetylases. Intriguingly, SCFAs have emerged as a crucial factor for the maturation and function of microglia, the resident macrophages of the central nervous system (CNS) [57]. Thus, the microglia of GF mice have an immature phenotype and exert impaired innate immune responses [58]. Importantly, the deficiency of SCFAs in GF mice and their actions on GPR43 (encoded by *Ffar2*) have been demonstrated to underlie these deficits. Thus, a 4-week treatment with SCFAs restores microglia malformation and immaturity in GF mice, whereas *Ffar2*^−/−^ mice show malformed microglia that is reminiscent of microglia in GF mice [57]. It is of particular note that no *Ffar2* mRNA expression was found on any cell type in the CNS (including microglia), whereas high expression was detected in the spleen. Therefore, direct actions of SCFAs on microglia are unlikely to mediate the observed effects, although SCFAs are able to enter the brain through transporters at the blood–brain barrier [26]. Determining the effects of SCFAs in *Ffar2*^−/−^ mice would give further insights into the mechanisms contributing to the SCFA-induced maturation of microglia.

As microglia, in addition to their immune function, are important for the shaping of neural circuits in the developing brain, their microbiota-dependent function could have a bearing on circuits regulating the HPA axis [59]. Interestingly, besides its influence on microglia, systemic administration of butyrate exerts antidepressant effects and modulates neurotransmission [28, 60, 61].

Apart from other functions, the neuropeptides GLP-1 and PYY, which are released by enteroendocrine cells in response to SCFAs, promote satiety through endocrine and vagus-dependent pathways [62, 63]. In addition to SCFAs, a recent study suggests that bacterial proteins may control appetite through local mechanisms in the gut or via the circulation [64]. The findings demonstrate that the nutrient availability stabilizes the exponential growth of *Escherichia coli* and that *E. coli* stationary-phase proteins are able to increase plasma PYY levels and suppress food intake when injected systemically [64]. Interestingly, the same laboratory had revealed before that *E. coli* protein caseinolytic protease B is an antigen-mimetic of α-melanocyte-stimulating hormone, a crucial satiogenic neuropeptide [65]. They further demonstrated that application of caseinolytic protease B to hypothalamic slices increases action potential frequency of pro-opiomelanocortin neurons, which produce α-melanocyte-stimulating hormone [64].

**Fig. 1:**
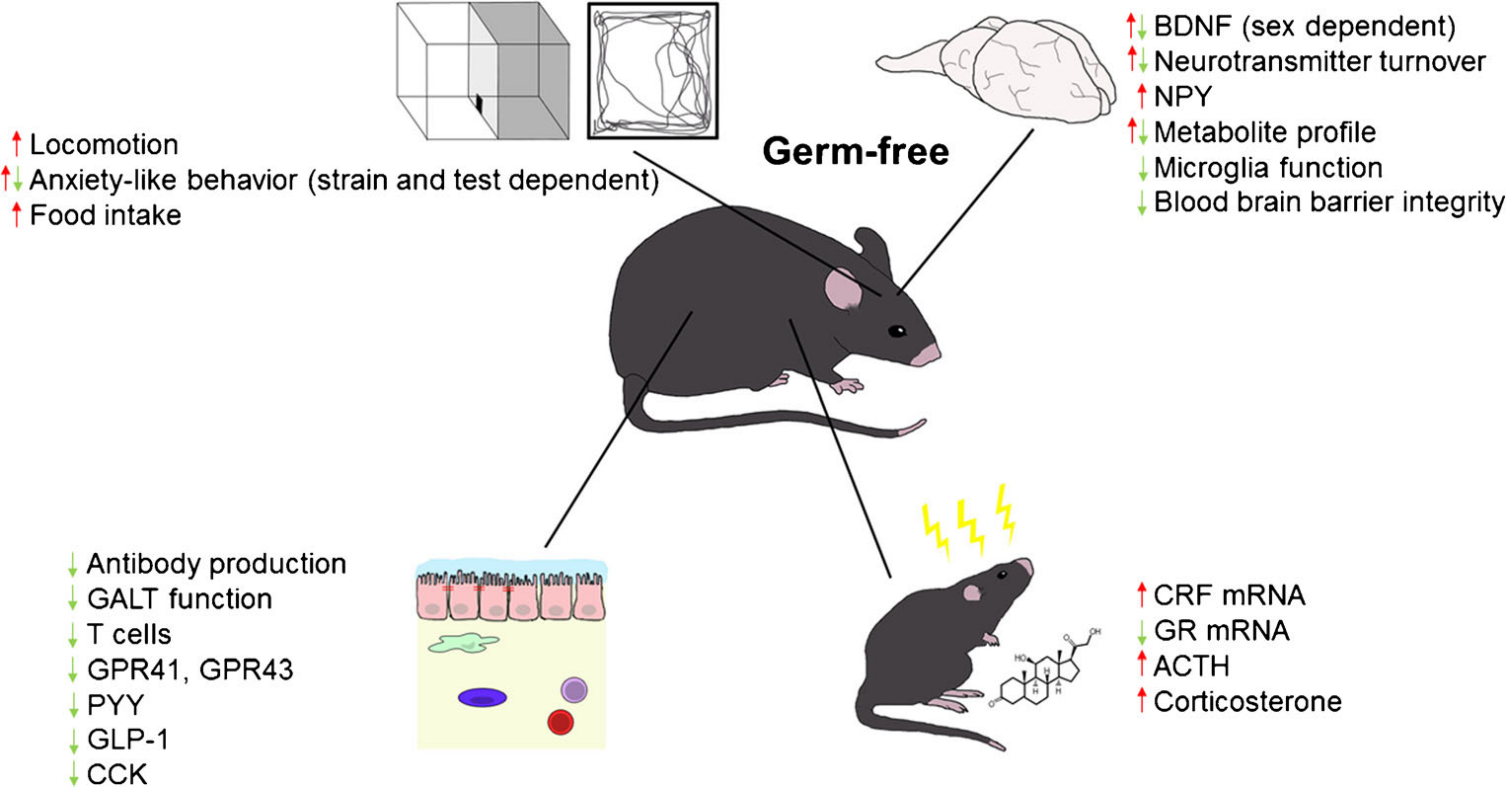
Neurodevelopmental changes in germ-free mice. Germ-free mice display developmental changes that affect various systems of the body that could impact on hypothalamus–pituitary–adrenal activity BDNF = brain-derived neurotrophic factor; NPY = neuropeptide Y; GALT = gut-associated lymphoid tissue; GPR = G protein-coupled receptor; PYY = peptide YY; GLP-1 = glucagon-like peptide 1; CCK = cholecystokinin; CRF = corticotropin-releasing factor; GR = glucocorticoid receptor; ACTH = adrenocorticotropic hormone

**Fig. 2:**
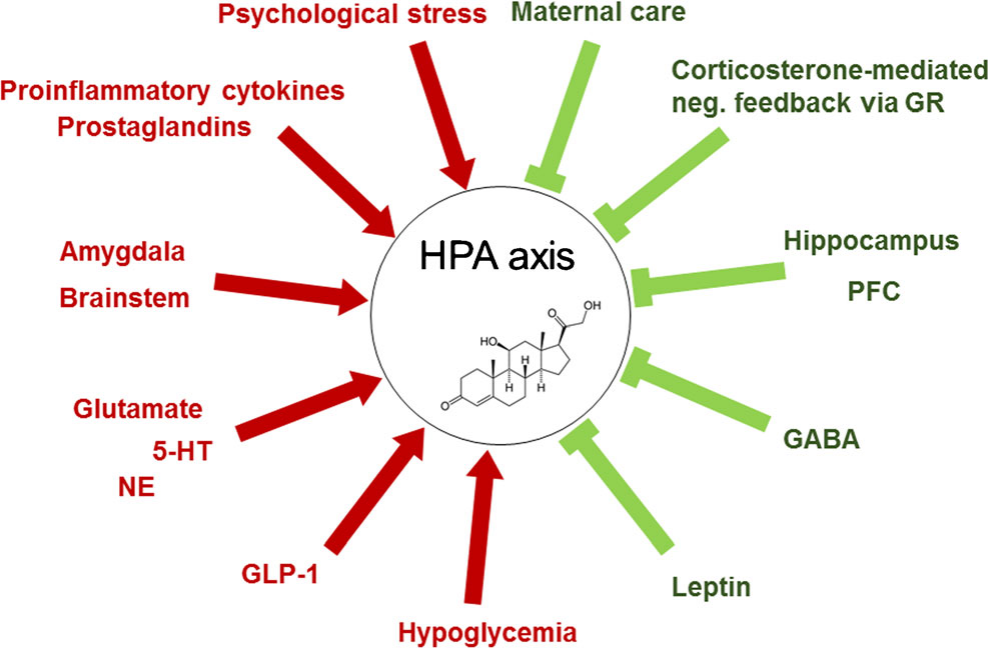
Modulators of the hypothalamus–pituitary–adrenal (HPA) axis. There are multiple activators (→) and inhibitors (˧) of the HPA axis GR = glucocorticoid receptor; PFC = prefrontal cortex; GABA = γ-aminobutyric acid; GLP-1 = glucagon-like peptide 1; NE = norepinephrine; 5-HT = 5-hydroxytryptamine

The expression of GPR41 and GPR43, as well as of PYY, GLP-1, and cholecystokinin, another intestinal satiety peptide is decreased in GF mice (Fig. 1) [66]. In addition, GF mice have lower levels of circulating leptin, decreased circulating glucose, and increased fat metabolism, a metabolic profile that resembles a fasting state [66]. In the hypothalamus of GF mice, the orexigenic neuropeptide Y (NPY) is increased, whereas anorexigenic neuropeptides are decreased [67]. The question therefore arises to which extent these differences contribute to the increased reactivity of the HPA axis of GF mice, given that apart from their effects on appetite these neuropeptides are able to affect behavior, brain function, and the neuroendocrine systems (Fig. 2) [25, 68].

In addition, the microbiota does not only control the release of various gut peptides, but also produces various neurotransmitters itself. For instance, *Lactobacillus brevis* and *Bifidobacterium dentium* are able to produce γ-aminobutyric acid (GABA) [69], whereas other bacterial species produce catecholamines [70]. While these neurotransmitters are likely to exert local effects, it still needs to be addressed whether these bacterial neurotransmitters would have any bearing on the gut–brain axis [71].

## Pro- and Prebiotics as Mostly Beneficial Modulators of the Neuroendocrine System

While an imbalance of the gut microbial community (induced by stress or diet) can lead to inflammatory processes and activation of the HPA axis, experimental work points to potential beneficial roles of probiotics, including lactobacilli and bifidobacteria in this process (Table 1).

**Table 1:** Selection of probiotics with reported preclinical effects on the neuroendocrine system

Probiotic/animal species	Additional intervention	HPA axis	Other effects	Behavioral effects	Reference
*Bifidobacterium pseudocatenulatum* (P2–P21) C57BL/6J ♂	MS (P2–P21)	↓CORT (P41) ┤MS-induced increase of CORT (P41)	↑TNF-α (gut, P21) ↓IFN-γ (gut, P21) ┤MS-induced increase of IL-18 (serum, P21) ┤MS-induced increase of Bacteroidetes (P21)	┤MS-induced anxiety (P41)	[76]
*B. pseudocatenulatum* (W7-21) C57BL/6 mice ♂	HFD (W7–W21)	↓CORT (W20) ┤Social stress-induced ↑CORT (W20) ┤HFD-induced decreased GR (W21)	┤HFD-induced increase of TLR2 (gut, hippocampus, W21) ↓ HFD-induced weight gain	┤HFD-induced anhedonia ┤HFD-induced anxiety (W18)	[73]
*Bifidobacterium infantis* (P50–P95) Sprague–Dawley rats ♂	MS (P2–P14)	↔CORT (P95)	┤Stress-induced increase of IL-6 (WBCS, ConA stimulated, P95)	┤MS-induced anhedonia (P90)	[74]
*Bifidobacterium longum* or *Bifidobacterium breve* (W8–W14) BALB/c mice ♂		↔CORT (W14)		↓ Anxiety (W12) ↓ Depression-like behavior (W13) ┤Stress-induced hyperthermia (*B. longum*, W11)	[75]
*Bifidobacterium animalis* subsp. lactis + *Propionibacterium jensenii* (P10–P22) Wistar rats	MS (P2–P14)	↑CORT (♀, P24) ↑ACTH (P24)	┤MS-induced decrease of IgA ┤MS-induced increase of *Escherichia coli*		[76]
*Lactobacillus rhamnosus* (W7–W11) BALB/c mice ♂		↓ Stress-induced CORT (W11)		↓ Anxiety (W10) ↓ Depression-like behavior (W10)	[77]
*Lactobacillus farciminis* (2 weeks) Wistar rats ♀	PRS	┤PRS-induced increase of CORT + ACTH (plasma) ┤PRS-induced increased CRF (mRNA, PVN)	┤PRS-induced increase of LPS (portal blood) ┤PRS-induced increase of IL-1β, IL-6 + TNF-α (mRNA, hypothalamus) ┤PRS-induced intestinal permeability		[80]
*Lactobacillus helveticus* + *B. longum* (2 weeks) C57BL/6 mice ♂	WAS	┤WAS-induced increase of CORT	┤WAS-induced c-Fos (PVN, amygdala, hippocampus) ┤WAS-induced decreased neurogenesis (hippocampus) ┤WAS-induced intestinal permeability		[81]
*L. rhamnosus* + *L. helveticus* (P4–P19) Sprague–Dawley rats	MS (P4–P19)	┤MS-induced increase of CORT	┤MS-induced intestinal permeability		[82]
*Lactobacillus fermentum* (P6–P21) Sprague–Dawley rats	MS (P10) WAS (P20)	┤MS-induced increase of CORT (P10) ┤WAS-induced increase of CORT (P20)	↓ Intestinal permeability (P20) ┤MS-induced intestinal permeability ┤WAS-induced intestinal permeability ↑ IFN-γ (activated splenocyte, P21)	↑ Locomotion	[83]

┤= prevention; ↓ = decrease; ↑ = increase; ↔ = no change; HPA = hypothalamic–pituitary–adrenal; P = postnatal day; B = Bifidobacterium; MS = maternal separation; CORT = corticosterone; TNF = tumor necrosis factor; IFN = interferon; IL = interleukin; HFD = high-fat diet; W = postnatal week; GR = glucocorticoid receptor; TLR = Toll-like receptor; WBCS = whole-blood culture supernatants; ConA = concanavalin A; ACTH = adrenocorticotropic hormone; L = Lactobacillus; PRS = partial restraint stress; PVN = paraventricular nucleus of the hypothalamus; WAS = water avoidance stress

A recent study showed that the probiotic *Bifidobacterium pseudocatenulatum* CECT 7765, administered from postnatal day 2 (P2) until P21, modified maternal separation (MS) stress-induced neuroendocrine alterations [72]. When tested at P21, *B. pseudocatenulatum* did not attenuate MS stress-induced increases in corticosterone levels, whereas stress-induced increases in catecholamines in the hypothalamus and small intestine were attenuated by the probiotic. However, at P41 *B. pseudocatenulatum* completely abolished stress-induced increases of corticosterone in stool and catecholamines in the hypothalamus, as well as anxiety-like behavior [72]. The potential involvement of immunomodulatory effects of *B. pseudocatenulatum* is suggested by a marked decrease of basal and stress-induced interferon (IFN)-γ levels in the small intestine, as well as of stress-induced interleukin (IL)-18 levels in the serum of probiotic-treated mice. In parallel to these immunomodulatory effects the probiotic prevented MS-induced increases of operational taxonomic units belonging mainly to the Bacteroidetes phylum [76].

Further studies by the same laboratory demonstrated that *B. pseudocatenulatum* CECT 7765 is able to attenuate various neuroendocrine alterations in response to administration of a HFD to adult male mice [73]. Again, the probiotic normalized HFD-induced fecal corticosterone levels (basal and in response to an acute stressor) and, in addition, reversed the tendency of HFD to decrease hippocampal GR levels. Moreover, *B. pseudocatenulatum* exerted anti-inflammatory effects and blunted HFD-induced behavioral and metabolic disturbances (Table 1) [73]. Finally, the probiotic affected DA, NE, epinephrine, and 5-hydroxytryptamine (5-HT) levels in the small intestine and brain. While the precise pathways of these probiotic effects have not been delineated, the data clearly show that *B. pseudocatenulatum* is able to ameliorate the effects of HFD-induced obesity, likely through anti-inflammatory actions and modulation of the neuroendocrine system.

While the studies referred to above point to the ability of certain strains of *Bifidobacterium* to affect the neuroendocrine system, other groups using different experimental protocols and different strains of *Bifidobacterium* did not see changes in corticosterone, although other parameters were affected. Thus, *Bifidobacterium infantis* 35624, given to maternally separated rats for 6 weeks from P50 to the day of sacrifice (P95), did not change baseline corticosterone levels, whereas stress-induced levels were not assessed [74]. However, *B. infantis* normalized MS-induced depression-like behavior, displayed certain anti-inflammatory effects, and affected central NE and 5-hydroxyindole acetic acid levels, the main metabolite of 5-HT [74]. Another study, conducted by the same group compared the effects of *Bifidobacterium longum* 1714 and *Bifidobacterium breve* 1205 in adult male BALB/c mice [75]. Again, while both probiotics displayed varying anxiolytic and antidepressant-like effects, they did not affect baseline or stress-induced corticosterone levels [75]. From these latter studies it could therefore be concluded that certain strains of *Bifidobacterium* exert particular behavioral effects that are not dependent on the neuroendocrine system. In contrast to these findings, the combination of *Bifidobacterium animalis subsp. lactis* BB-12 and *Propionibacterium jensenii* 702 (a probiotic isolated from dairy) administered to Wistar rats 10 days before conception until weaning increased corticosterone levels in female offspring [76].

Various groups have also investigated the central and neuroendocrine effects of different strains of *Lactobacillus.* In particular, the finding that chronic treatment of nonstressed adult male BALB/c mice with the probiotic *Lactobacillus rhamnosus* (JB-1) for 28 days is able to decrease corticosterone levels, reduce depressive-like behavior in the forced swim test, and decrease anxiety-like behavior on the elevated plus maze (EPM), attracted a lot of attention within and outside the scientific community [77]. However, these effects of *L. rhamnosus* (JB-1) appear to be strain- and species-dependent, given that the probiotic fails to affect the baseline social and anxiety-like behavior in male C57BL/6 mice, whereas it did attenuate stress-induced behavioral disturbances [78]. Likewise, an 8-week intake of *L. rhamnosus* (JB-1) by 29 healthy male subjects did not alter salivary cortisol, mood, anxiety, sleep quality, subjective stress, visuospatial memory performance, attention switching, rapid visual information processing, emotion recognition, and associated electroencephalography parameters [79].

As the behavioral effects in response to *L. rhamnosus* in BALB/c mice are not present in vagotimized mice, the vagus nerve is suggested to be a critical pathway of communication between probiotic signaling and the CNS [77]. Likewise, vagotomy prevented some changes in central GABA receptor expression induced by *L. rhamnosus*, whereas corticosterone levels in vagotomized mice were not reported [77].

In addition to these emotional-affective and neurochemical changes, lactobacilli have been repeatedly reported to exert beneficial effects in various stress protocols (Table 1). A 2-week treatment of female Wistar rats with *Lactobacillus farciminis* followed by partial restraint stress is able to block stress-induced increases in ACTH and corticosterone, as well as CRF in the PVN [80]. In addition, the probiotic was able to inhibit stress-induced hyperpermeability of the intestinal barrier, as well as increased LPS levels in the portal blood. Consistent with these effects, *L. farciminis* prevented stress-induced increases of IL-1β, IL-6, and tumor necrosis factor (TNF)-α mRNA expression in the hypothalamus [80]. In a subsequent study the same group reported that a 2-week pretreatment with a probiotic formulation containing *Lactobacillus helveticus* R0052 and *B. longum* R0175 blunts increased levels of corticosterone, NE, and epinephrine of adult male mice in response to water avoidance stress (WAS) [81]. These effects were paralleled by prevention of gut barrier impairment in response to stress, reduced neuronal activity (c-Fos expression) in stress-sensitive brain areas, including the PVN and amygdala, and an increase in hippocampal neurogenesis in control and stressed animals. In addition, the probiotic formulation affected hypothalamic genes involved in synaptic plasticity in stressed mice, increasing the expression of brain-derived neurotrophic factor (BDNF), while decreasing the expression of microglial activation markers [81]. Similarly, the probiotic combination of *L. rhamnosus* R0011 and *L. helveticus* R0052, administered to maternally separated rat pups, attenuated the increased corticosterone levels, as well as the increased permeability of the colonic barrier [82]. Furthermore, administration of *Lactobacillus fermentum* CECT5716 to newborn rats has been recently reported to reduce intestinal permeability *in vivo* under basal conditions and in response to MS or WAS [83]. The rise of corticosterone levels in response to MS or WAS was attenuated by the probiotic. Moreover, the probiotic increased IFN-γ [a marker of T helper (Th)1 response] secretion of activated splenocytes, whereas IL-4 (a marker of Th2 response) secretion was inhibited. Finally, *L. fermentum* increased locomotion and exploratory behavior [83].

As it has been suggested that a combination of several probiotic species may exert additive effects, a recent study investigated the interaction of a HFD (starting at 4 weeks of age) with a multispecies probiotic formulation (starting at 9 weeks of age) containing 8 bacterial strains (*Bifidobacterium bifidum* W23, *Bifidobacterium lactis* W52, *Lactobacillus acidophilus* W37, *L. brevis* W63, *Lactobacillus casei* W56, *Lactobacillus salivarius* W24, *Lactococcus lactis* W19, *Lactococcus lactis* W58) in male Sprague–Dawley rats [84]. While the probiotic formulation did not affect HFD-induced increase of plasma LPS levels and metabolic changes, the formulation exerted various diet-independent effects. Thus, probiotic treatment reduced depression-like behavior and hippocampal mRNA expression of CRF receptor (CRFR)1 and CRFR2, indicating a potentially reduced activity of the HPA axis. In keeping with other reports, the probiotic formulation modulated the cytokine production by stimulated blood mononuclear cells towards T lymphocyte-derived cytokines (IFN-γ, IL-2, IL-4), whereas macrophage-associated cytokines (TNF-α, IL-6) were reduced. Furthermore, the probiotic formulation increased the levels of indole-3-propionic acid, a microbial tryptophan metabolite that has been demonstrated to be neuroprotective and reduce CNS inflammation [85].

The findings of predominantly beneficial effects of probiotics in rodents are matched by some positive effects of different probiotic strains in humans. For instance, in a randomized, double-blind, placebo-controlled study, *Lactobacillus plantarum* 299v was administered to 41 students with an upcoming exam and associated with decreased corticosterone levels after 10 days [86]. Similarly, a probiotic formulation consisting of *L. helveticus* R0052 and *B. longum* R0175 (which had been effective in mice), decreased median urinary free cortisol levels and psychological distress, when given to 26 healthy volunteers for 30 days in a double-blind, controlled, randomized, parallel study [87]. Similarly, *B. longum* 1714 given to 22 healthy male volunteers in a placebo-controlled study attenuated increases in cortisol levels and subjective anxiety in response to an acute stressor [88]. These effects were paralleled by improvements in hippocampus-dependent visuospatial memory performance and changes in brain activity, as assessed by electroencephalography [88].

In addition to probiotics, prebiotics—non-digestible fiber compounds that stimulate the growth of beneficial bacteria—have also been reported to exert positive effects on the microbiota–gut–brain axis. A recent study compared the effects of a 3-week administration of the prebiotics fructo-oligosaccharides (FOS), galacto-oligosaccharides (GOS), and their combination in adult male mice [89]. Prebiotic administration reduced stress-induced plasma corticosterone levels, with GOS and the combination of FOS and GOS having the strongest effects and attenuated hippocampal mRNA expression of CRFR1. In line with this finding, GOS and FOS + GOS administration reduced anxiety and depression-like behavior. In particular, the combination of FOS + GOS increased the expression of BDNF and the GABA_B_ receptor in the hippocampus and 5-HT levels in the prefrontal cortex. Prebiotics also induced changes in gut microbiota composition, with surprising decreases of the relative abundance of lactobacilli and bifidobacteria. In contrast, the cecal SCFAs acetate and propionate were increased, whereas those of isobutyrate were decreased by the prebiotics. Remarkably, the changes in SCFA levels correlated with the behavioral alterations. In addition to these baseline effects, a 3-week administration of FOS + GOS was demonstrated to blunt the effects of chronic social stress in increasing corticosterone levels and spleen cytokine production in response to stimulation with concanavalin A and in exacerbating stress-induced behavioral disturbances [89]. In line with these beneficial effects in mice, a clinical study reported that GOS, but not FOS, are able to decrease the salivary cortisol awakening response in healthy volunteers [90].

It should not go unnoticed, however, that pre- and probiotics may also have adverse effects on HPA axis and behavior. Thus, a recent study administering *L. casei* 54-2-33, the prebiotic inulin, or a mixture of the probiotic and prebiotic (synbiotic) to male Sprague–Dawley rats starting at P21 for 14 days, found increased baseline corticosterone levels in response to the probiotic or prebiotic alone, whereas the synbiotic attenuated stress-induced increases of corticosterone [91]. Surprisingly, all treatments had anxiogenic effects in the open-field test (OFT), whereas the synbiotic exerted anxiolytic effects in the EPM test [91].

## Gut Microbiota and the HPA Axis: Evidence from GF Mice

Studies performed in mice that have been raised within GF isolators and are therefore devoid of any microorganisms have been of substantial use in further delineating a role of the gut microbiota in shaping the neuroendocrine system (Table 2). Thus, GF mice have been demonstrated to have increased CRF expression, reduced GR expression in the cortex, and elevated plasma ACTH and corticosterone levels in response to restraint stress (Table 2) [93, 95]. Colonization of neonatal GF mice with *B. infantis* is able to attenuate the increased responsiveness of the HPA axis in GF mice, whereas colonization with enteropathogenic *E. coli* exacerbates the HPA response to restraint stress [93]. While the precise pathways have not been elucidated, changes in immune-mediated effects on the HPA axis have been suggested to play a significant role. Thus, while both *E. coli* and *B. infantis* induce increases in plasma corticosterone and c-Fos mRNA levels in the PVN 6 h, and a slight increase of IL-6 levels 12 h, after inoculation, these effects are more pronounced and longer lasting in response to inoculation with *E. coli.* Furthermore, only *E. coli* leads to an increase of IL-1β in plasma, with highest levels 12 h after inoculation [93]. Colonization of GF mice with feces of specific-pathogen-free (SPF) mice at the age of 6 weeks is able to prevent the exacerbated HPA response, whereas colonization at the age of 14 weeks does not affect the HPA axis, indicating age-dependent effects of bacterial colonization at a critical developmental window [93].

**Table 2:** Changes in neuroendocrine system of germ-free (GF) mice

Mouse strain	HPA axis (GF *vs* colonized)	Related systems	Reference
GF BALB/c mice ♂	↑ CORT (after restraint stress) ↑ CRF mRNA + protein (hypothalamus) ↓ GR mRNA (cortex)	↓ NR-1 mRNA (cortex) ↓ NR-2 mRNA (cortex + hippocampus) ↓ BDNF protein (cortex + hippocampus)	[93]
GF BALB/c mice		↓ NE and DA turnover (hippocampus, striatum, brainstem) ↓↑ 5-HT turnover (striatum, brainstem)	[101]
GF Swiss Webster mice ♀	↑ CORT (48 h after arrival)	↓ NR2b mRNA (amygdala) ↑ BDNF mRNA (hippocampus) ↓ 5HT_1A_ mRNA (hippocampus)	[98]
GF NMRI mice ♂		↑ NE, DA, and 5-HT turnover (striatum) ↓ NGFI-A mRNA (PFC) ↓ BDNF mRNA (hippocampus + amygdala) ↑ DA_D1_ receptor mRNA (hippocampus) ↑ synaptophysin + PSD-95 (striatum)	[97]
GF Swiss Webster mice ♀+♂	↑ CORT (after novel environment stress)	↓ BDNF mRNA (hippocampus, male) ↑ 5-HT (hippocampus, male) ↑ 5-HIAA (hippocampus, male) ↓ TNF-α (splenocytes + LPS) ↓ kynurenine/tryptophan (plasma)	[99]
GF stress-sensitive F344 rats ♂	↑ CORT after OFT ↑ CRF mRNA (PVN) ↓ GR mRNA (hippocampus)	↓ DA turnover (frontal cortex, hippocampus, striatum)	[102]
GF C57BL/6N mice ♀+♂	↔ CORT after MS	↑ BDNF protein after MS (hippocampus) ↑ 5-HT (hippocampus) ↓ NE (hippocampus)	[100]

HPA = hypothalamic–pituitary–adrenal; CORT = corticosterone; CRF = corticotropin-releasing factor; GR = glucocorticoid receptor; NR = N-methyl D-aspartate receptor; BDNF = brain-derived neurotrophic factor; NE = norepinephrine; DA = dopamine; 5-HT = 5-hydroxytryptamine; NR2b = 2b subunit of the *N*-methyl-D-aspartate receptor; NGFI-A, nerve growth factor-inducible clone A; PFC = prefrontal cortex; PSD-95 = postsynaptic density protein 95; 5-HIAA = 5-hydroxyindole acetic acid; TNF = tumor necrosis factor; LPS = lipopolysaccharide; OFT = open-field test; PVN = paraventricular nucleus of the hypothalamus; MS = maternal separation

GF mice present with many neuroendocrine and neurochemical alterations that provide hints at a possible involvement of the microbiota. For instance, there is evidence for a decreased central gene expression of the NMDA receptor subunits and BDNF in GF mice (Table 2) [93]. Both BDNF and NMDA receptor subunits play a role as mediators of synaptic plasticity and changes in their expression have been linked to psychiatric disorders [92, 94, 95]. Interestingly, decreases in hippocampal BDNF, as well as glutamate NMDA receptor subunits, are induced by maternal deprivation and have been proposed to contribute to long-term impairment in brain function [96]. Decreased BDNF levels in the hippocampus and amygdala of GF mice are associated with decreased expression of nerve growth factor-inducible clone A, whereas postsynaptic density protein 95 and synaptophysin, markers that are associated with synaptogenesis and maturation, are increased in GF mice. In addition, the turnover of NE, DA, and 5-HT is increased, as is DA_D1_ receptor mRNA [97].

Neufeld et al. [98] reported increased corticosterone levels in female GF mice. In contrast to the decreased BDNF levels in the hippocampus of male mice, they observed increased BDNF mRNA in the hippocampus of female mice. Clarke et al. [99] also compared male and female GF mice, and could observe marked sex differences in the neurochemical parameters, whereas the neuroendocrine and immunologic parameters did not differ between the sexes. In line with the aforementioned studies, they demonstrated that male GF mice display decreased BDNF levels in the hippocampus, whereas the BDNF levels in female mice tended to be slightly increased. Sex differences were also seen for hippocampal 5-HT and its main metabolite 5-hydroxyindole acetic acid, as increased levels were only seen in male GF mice. In contrast, corticosterone levels in response to novel environment stress were exaggerated in both male and female mice [99]. In an attempt to establish a potential link between the immune and neuroendocrine system, cytokine release from splenocytes following stimulation with LPS was found to be blunted in GF mice. As the decrease in cytokine formation was unrelated to sex, one could speculate that the increase in plasma corticosterone is rather linked to the immunologic but not neurochemical changes in GF mice.

A study investigating MS as a model of early life stress concluded that alterations of the HPA axis in response to MS are independent of the gut microbiota, whereas the behavioral disturbances are related to dysbiosis of the gut microbiota [100]. This conclusion was based on the finding that the MS-induced elevation of corticosterone levels was comparable in GF and SPF mice, whereas anxiety-like behavior and behavioral despair were only induced by MS in the presence of gut microbiota [100].

In summary, it can be concluded that increased reactivity of the HPA axis is frequently found in GF mice. In contrast to other parameters, such as neurochemical changes and behavior, which vary dependent on sex, mouse strain, and other factors, increased reactivity of the HPA axis is frequently found in GF mice and rats, although only a limited number of GF mouse strains have so far been tested (Table 2).

While various groups have demonstrated increased sensitivity of the HPA axis in GF mice, together with changes in neurocircuits linked to the HPA axis, the direction of this interaction is not clear. The HPA axis is in mutual relationship with neuronal systems in limbic, midbrain, and brainstem nuclei, as well as with the sympathetic and parasympathetic nervous system [3]. Thus, while modulation of stress hormones by the lack of gut microbiota could potentially induce changes in neurotransmitter systems, other factors could have an impact on neurocircuits that subsequently affect the HPA axis. In this regard, developmental defects of the immune system and changes in metabolism of GF mice are likely to contribute to the neuroendocrine phenotype in the absence of gut microbiota.

## Neuroendocrine-Associated Behavioral Phenotype of GF Mice

In line with the increased responsiveness of the HPA axis of GF mice, GF BALB/c mice have been demonstrated to exhibit increased anxiety-like behavior in the OFT, as well as increased spontaneous motor activity [101]. However, 2 other reports found a decrease in anxiety-like behavior of GF mice when tested in the light/dark box or the EPM test, despite an increased responsiveness of the HPA axis [97, 98]. The inconsistency of findings is further illustrated by a report of both increased and decreased anxiety-like behavior in GF mice depending on the behavioral test (step-down test and light preference, respectively) [100]. Finally, a study conducted in male F344 rats found increased anxiety-like behavior in the OFT [102]. Therefore, it can be concluded that the behavioral test employed, the animal species tested, and the genetic background have an impact on the behavioral phenotype of GF rodents. It appears as if stress-sensitive strains such as BALB/c mice and F344 rats display increased anxiety under GF conditions [101, 102], whereas moderately emotive strains such as Swiss and NMRI mice do not [97, 98]. In addition, the behavioral differences of different mouse strains are also affected by the gut microbiota [103]. Thus, the transfer of the microbiota from BALB/c mice to GF NIH Swiss mice decreases exploratory behavior *versus* colonization with NIH Swiss microbiota, whereas colonization of BALB/c mice with microbiota of NIH Swiss mice increases their exploratory behavior [103]. Another potential confounding factor in the assessment of anxiety-like behavior in GF mice could be hyperlocomotion, which has been consistently observed in GF mice [98, 101] and GF zebrafish [104]. Thus, increased locomotion is able to interfere with the evaluation of anxiety and depression-like behaviors, which may be incorrectly interpreted as paradoxical “anxiolytic-like” behavioral patterns [105, 106].

## Towards Potential Mechanisms: Developmental Defects of GF Mice

Apart from changes in the neuroendocrine system, GF mice show developmental deficits of the gastrointestinal tract (Fig. 1) [107]. Interestingly, the severity of animal models of Th17 cell-dependent arthritis and experimental autoimmune encephalomyelitis is markedly attenuated when tested in GF mice [108, 109]. In contrast, GF mice have higher numbers of invariant natural killer T cells in the colonic lamina propria and are more susceptible to inflammatory conditions that are dependent on invariant natural killer T cells, such as oxazolone-induced colitis [110]. A role of the microbiota in the maturation of the immune system is indicated by the observations that colonization of neonatal GF mice with a conventional microbiota is able to prevent oxazolone-induced colitis, whereas colonization of adult GF mice does not [110].

It is not clear to which extent the deficits in lymphocyte differentiation could contribute to the neuroendocrine disturbances in GF mice. Interestingly, however, recombinase activator gene 1-deficient mice, which lack mature B and T lymphocytes, display impaired nonspatial memory and increased anxiety-like behavior, as well as increased corticosterone levels [111]. In addition, administration of a probiotic mixture containing *L. rhamnosus* and *L. helveticus* from weaning onwards is able to ameliorate the behavioral deficits and to partly restore gut microbiota dysbiosis, whereas corticosterone levels remain elevated [111]. In addition, GF mice present with various functional and biochemical alterations of the brain [57, 112–114], which may also be factors in the development of neuroendocrine and behavioral disturbances. Thus, genome-wide transcriptional profiling of the amygdala of GF mice revealed features of increased neuronal activity, whereas immune system-related genes were downregulated [115]. Furthermore, GF mice have an enlarged amygdala and hippocampus associated with dendritic hypertrophy of subsets of amygdalar neurons, whereas hippocampal neurons showed dendritic atrophy [116]. Post-transcriptional gene expression has also been demonstrated to be controlled by the gut microbiota as GF mice show an altered microRNA (miRNA) expression profile in the amygdala and prefrontal cortex [117]. Interestingly, a role of the miRNA miR-21-5p has also been demonstrated for microbiota-induced increase of intestinal epithelial permeability through upregulation of the small guanosine triphosphatase adenosine diphosphate ribosylation factor 4 [118].

## Towards Potential Mechanisms: Metabolic Deficiencies in GF Mice

The gut microbiota makes an important contribution to the host’s digestion and metabolism, and is an important supplier of vitamins [119]. Thus, GF mice require exogenous vitamin K and vitamin B in their diet [119]. The question therefore arises as to whether the brain’s high metabolic demand is insufficiently covered under conditions of a missing or dysbiotic microbiota, especially during sensitive periods, such as in early life, and how these potential deficiencies could contribute to long-term neuroendocrine disturbances, given that dietary deficiencies are known to affect the HPA axis [120–122]. Interestingly, while the polyunsaturated fatty acid eicosapentaenoic acid is not detectable in GF mice [114], long-term supplementation with a polyunsaturated fatty acid mixture that includes eicosapentaenoic acid reverses MS-induced dysbiosis and attenuates the corticosterone response to acute stress [123]. In contrast, free fatty acids have been reported to activate and inhibit the HPA axis in rats and humans, respectively [124, 125].

There are many other metabolite changes in the blood of GF mice. For instance, the bacterially mediated production of bioactive indole-containing metabolites that derive from tryptophan is impaired, whereas the availability of tryptophan, which is a precursor of 5-HT, is increased [114]. While Clarke et al. [99] found increased 5-HT levels in the hippocampus of male, but not female, GF mice, metabolomic assessment of the prefrontal cortex of male GF mice did not reveal any differences in 5-HT levels [113]. In contrast, metabolites involved in glycolysis pathways were changed in GF mice, which suggests that GF mice consume less energy through glycolysis than conventionalized mice. Unlike cerebral GABA, which was not altered, the concentration of DA was twofold higher in GF mice and has been suggested to underlie the increased motor activity seen in these animals [113].

## Antibiotic-Induced Manipulation of the Gut Microbiota and Neuroendocrine System

In view of the various systems that are affected in their development when mice are raised GF, antibiotic-induced dysbiosis represents an alternative way to manipulate the microbiota in adult animals without inducing developmental changes and with the advantage of choosing the time point and severity of the dysbiosis caused. As the composition of the gut microbiota can be altered by stress itself [126], we first discuss the effects of antibiotic-induced gut dysbiosis per se on neuroendocrine, neurochemical, and behavioral parameters, followed by a discussion of the effects of dysbiosis in conjunction with different acute and chronic stress protocols.

## Antibiotic-Induced Gut Dysbiosis: Effects on Neuroendocrine, Neurochemical, and Behavioral Parameters

Early dysbiosis disturbing the establishment of a mature microbiota could have long-lasting effects on the microbiota, the development of the HPA axis, and the overall health of the host (Table 3). In line with findings in GF animals, several studies demonstrate increased corticosterone levels in response to antibiotic-induced gut dysbiosis. To investigate the role of early-life antibiotic treatment, Scheer et al. [127] exposed mice to an antibiotic cocktail containing ampicillin, streptomycin, vancomycin, and metronidazole, and the artificial sweetener sucralose from gestation until weaning (P21). While serum corticosterone levels were similar in control and antibiotic-treated pups at P7, serum corticosterone levels tended to be increased in antibiotic-treated mice at P21 and P42. Interestingly, co-housing of antibiotic-treated mice with control animals 1 week after weaning (P28) was able to reverse the increase of serum corticosterone levels as assessed at P56. Although the gut microbiota composition was not investigated in this study, this finding could suggest that antibiotic-induced changes in gut microbiota composition and subsequent effects on the HPA axis can be reversed by co-housing through normalization of the gut microbiota composition. In addition to the increased corticosterone levels, CD4^+^ T cells from antibiotic-treated mice showed altered expression of stress response-, cellular metabolism-, cell-cycle regulation-, and cell death-related genes and induced an earlier onset of colitis, when transferred to recombinase activator gene 1-deficient mice, which develop colitis in response to CD4^+^ T cells owing to their defects in the immunoregulation of mucosal T-cell responses [128].

**Table 3:** Antibiotic-induced dysbiosis models and their impact on the neuroendocrine system

Antibiotics	Administration duration	Animal model	Stress model	HPA axis	Reference
AMP (0.5 g/l), streptomycin (0.5 g/l), vancomycin (0.5 g/l), metronidazole (0.5 g/l), splenda (4 g/l)	Drinking water; during gestation and until weaning (day 21)	Foxp3 reporter mice on C57BL/6 background; sex not listed	No stress	↔CORT (day 7, serum, thymus, proximal colon, cecum) (↑)CORT (day 21 and 42, serum) ↑CORT(day 21 and 42, thymus, proximal colon, cecum)	[127]
Bacitracin (24 mg/ml), neomycin (24 mg/ml), AMP (9.6 mg/ml), meropenem (4.8 mg/ml), vancomycin (1.44 mg/ml)	Oral gavage; 10 days	C57BL/6N mice; ♂	No stress	↑CORT (plasma, after last day of treatment)	[129]
AMP (120 mg/kg) or AMP(120 mg/kg) and *Lactobacillus fermentum* NS9 (AMP light phase, water/NS9 dark phase)	Drinking water; 41 days	Sprague–Dawley rats; ♂	No stress	↑CORT (AMP, serum, after MWM on the last day of treatment) ↔GR (HIP) ↓MR (AMP, HIP)	[131]
Vancomycin 10, 30, or 100 mg/kg	Oral gavage; 10 days	Sprague–Dawley rats; ♂	No stress	↔CORT (plasma, 10 weeks after treatment)	[132]
0.5% neomycin and 1% AMP	Drinking water; 12 days	Wistar rats; ♀	Acute partial restraint stress (2 h)	┤CORT (plasma, immediately after stress) ┤CRF mRNA (HYP)	[80]
AMP (1 mg/ml), vancomycin (5 mg/ml), neomycin (10 mg/ml), metronidazole (10 mg/ml), amphotericin B (0.1 mg/ml)	Drinking water; 59 days	NIH Swiss mice; ♂	Acute restraint stress (30 min)	↔CORT (plasma, immediately after stress) ↔CRF mRNA (HYP)	[133]
AMP (1 g/l), vancomycin (500 mg/l), ciprofloxacin HCl (20 mg/l), imipenem (250 mg/l), metronidazole (1 g/l)	Drinking water; 13 weeks	Sprague–Dawley rats; ♂	Acute FST	↔CORT (during FST) ↓NR3C1 mRNA (AMY, HIP) ↔NR3C2 mRNA (AMY, HIP) ↓CRFR1 mRNA (AMY, HIP) ↔CRFR2 mRNA (AMY, HIP) 3 weeks after FST	[134]
Streptomycin sulfate (2 mg/ml) and penicillin G (1500 U/ml)	Drinking water; 21 days	Sprague–Dawley rats; ♂	Psychological stress—chronic mild stress (21 days)	┤CORT (plasma, immediately after stress)	[135]
Minimum dose: 0.4 mg bacitracin, 0.4 mg neomycin, 0.1 mg amphotericin B per mouse/day	Oral gavage and drinking water; 7 days	C57BL/6N mice; ♀	Repetitive psychological stress—WAS (1 h/day, 7 days)	↔CORT (immediately after stress) ↔Weight of the adrenal glands	[136]

↔ = no change; ┤= prevention of stress-induced increase; ↓ = decrease; ↑ = increase; () = trend; HPA = hypothalamus–pituitary–adrenal; CORT = corticosterone; NS9 = *Lactobacillus fermentum* NS9; AMP = ampicillin; MWM = Morris water maze; GR = glucocorticoid receptor; HIP = hippocampus; MR = mineralocorticoid receptor; CRF = corticotropin-releasing hormone; HYP = hypothalamus; FST = forced swim test; NR3C1 = nuclear receptor subfamily 3, group C, member 1 (glucocorticoid receptor); NR3C2 = nuclear receptor subfamily 3, group C, member 2 (mineralocorticoid receptor); AMY = amygdala; CRFR1 = corticotrophin-releasing hormone receptor 1; CRFR2 = corticotropin-releasing hormone receptor 2; WAS = water avoidance stress

Treatment of adult male mice with an antibiotic cocktail (ampicillin, bacitracin, meropenem, neomycin, vancomycin) for 10 days has been demonstrated to severely disrupt the microbial composition (16S rDNA) in the colon and to affect various levels of the gut–brain axis [129]. Again, basal corticosterone levels in the plasma were increased in antibiotic-treated animals and nonspatial memory were impaired by the antibiotic treatment. Furthermore, SCFA levels in the colonic contents were decreased, and the levels of lipid species and converted bacteria-derived metabolites in the plasma were changed in antibiotic-treated mice. Gene expression in the brain of antibiotic-treated animals was modified in a region-specific manner. In the amygdala and hypothalamus, NPY mRNA expression—an orexigenic neuropeptide promoting stress resilience [130]—was increased in antibiotic-treated mice, whereas NPY receptor expression was decreased in the amygdala and the hippocampus. Expression of the 5-HT transporter and a subunit of the NMDA receptor was increased in the amygdala of antibiotic-treated animals while expression of BDNF mRNA was decreased in the medial prefrontal cortex, hippocampus, and hypothalamus. Whereas cytokine levels in the blood were not increased by antibiotic treatment, mRNA expression of IL-1β was decreased in the hippocampus and hypothalamus. In addition, the mRNA expression patterns of 3 tight junction proteins in the amygdala and hippocampus were affected by antibiotic treatment [129]. These findings suggest that the increase in HPA axis activity associated with antibiotic-induced gut dysbiosis may be related to complex changes in gut–brain interaction.

Treatment with only ampicillin has been shown to appreciably alter fecal microbiota composition (assessed by quantitative polymerase chain reaction) in rats and to enhance serum corticosterone levels [131]. When rats were treated with ampicillin in the light phase and with water or the probiotic *L. fermentum* NS9 (NS9) in the dark phase, the antibiotic-evoked rise of serum corticosterone was reversed by NS9. While the levels of GR and BDNF in the hippocampus stayed unchanged, the levels of mineralocorticoid and NMDA receptors were decreased in antibiotic-treated rats. Treatment with NS9 was likewise able to normalize the levels of NMDA and mineralocorticoid receptors in the hippocampus. At the behavioral level, antibiotic treatment alone induced mild anxiety-like behavior and a deficit in spatial memory retention; these disturbances were prevented by co-treatment with the probiotic NS9 [131]. In contrast to the mentioned studies, treatment of male rat pups with different concentrations of vancomycin from P4 to P13 did not affect basal levels of corticosterone in the plasma in adulthood [132], whereas visceral sensitivity and pain-related behavior were increased by this antibiotic treatment in a dose-dependent manner. Furthermore, in rats receiving the highest dose of 100 mg/kg vancomycin, basal levels of IL-6 and neutrophils were increased in splenocytes, whereas no differences in cytokine levels in whole blood were found [132].

## Antibiotic-Induced Dysbiosis in Combination with Acute or Chronic Stressors

Antibiotic-induced dysbiosis studies investigating the effect of acute stress on the HPA axis offer the opportunity to measure basal and stress-induced corticosterone levels. Ait-Belgnaoui et al. [80] showed that antibiotic treatment (ampicillin, neomycin) for 12 days prevented the augmentation of corticosterone levels in the plasma of female rats subjected to acute partial restraint stress (2 h). Furthermore, antibiotic treatment was able to reverse the stress-induced increase in CRF, IL-1β, IL-6, and TNF-α mRNA expression in the hypothalamus. While gut dysbiosis was not examined, the concentration of LPS in the portal blood was significantly reduced in antibiotic-treated animals [80]. Desbonnet et al. [133] investigated an antibiotic-induced dysbiosis model where animals were treated from weaning onwards and then subjected to acute restraint stress (30 min) immediately before sacrifice. Antibiotics blunted the stress-induced increase of bacterial number and diversity. While antibiotic treatment neither affected hypothalamic CRF mRNA expression nor altered the plasma levels of corticosterone levels at baseline and following acute restraint stress, changes in behavior and HPA axis-related systems were observed [133]. Thus, expression of hippocampal BDNF mRNA and hypothalamic vasopressin mRNA were reduced by antibiotic treatment [133]. Antibiotic treatment further increased the levels of NE in the hippocampus, as well as L-3,4-dihydroxyphenylalanine and the DA metabolite homovanillic acid in the amygdala [133]. In another study, adult male rats were treated for 13 weeks with an antibiotic cocktail (ampicillin, vancomycin, ciprofloxacin, imipenem, metronidazole), during which the animals were subjected once to the forced swim test as an acute stressor [134]. While long-term antibiotic exposure did not alter basal and stress-induced corticosterone levels, stress-induced fecal output was increased in antibiotic-treated animals. The levels of GR mRNA and CRFR1 mRNA were decreased in the amygdala and hippocampus of antibiotic-treated rats, whereas BDNF mRNA expression was increased in the amygdala of antibiotic-treated rats [134]. At the behavioral level, antibiotic treatment increased depression-like behavior and caused spatial memory deficits and affected monoamine levels in several brain regions [134].

A study investigating the effects of antibiotic treatment (penicillin G, streptomycin sulfate) during exposure to chronic mild stress (21 days) observed that antibiotics were able to prevent the stress-induced increase in corticosterone levels in the plasma of adult male rats [135]. Moreover, in stressed animals, antibiotic treatment had an anti-inflammatory effect in the brain as the stress-induced rise in cortical inflammatory mediator levels (including cyclooxygenase-2, IL-1β, and prostaglandin E_2_) was prevented by antibiotic treatment. In keeping with these anti-inflammatory effects, antibiotic treatment also blocked the stress-induced decrease of the anti-inflammatory mediator 15-deoxy-delta^12,14^-prostaglandin J_2_ in the prefrontal cortex. The stress-induced alterations of cortical nuclear factor kappa B levels were also prevented by antibiotic treatment. Interestingly, depression-like behavior was not affected by antibiotic treatment, but the number of fecal boli excreted during the modified forced swim test was increased in antibiotic-treated mice independently of the stress protocol [135]. Gut dysbiosis was not examined, but the stress-induced increase in LPS and LPS binding protein concentrations in the plasma was prevented in antibiotic-treated animals. In keeping with these results, the stress-induced rise of TLR4 levels in the prefrontal cortex was blocked by antibiotic treatment [135].

Another study using WAS as a repetitive psychological stressor (7 days) showed that antibiotic-induced dysbiosis does not alter endocrine responses to chronic stress but prevents the enhanced visceral pain-related response seen in stressed female mice [136]. While several inflammatory markers (IL-6, TNF-α) stayed unaltered, secretory immunoglobulin A levels in the cecal lumen were increased in antibiotic-treated animals independently of stress [136].

**Fig. 3:**
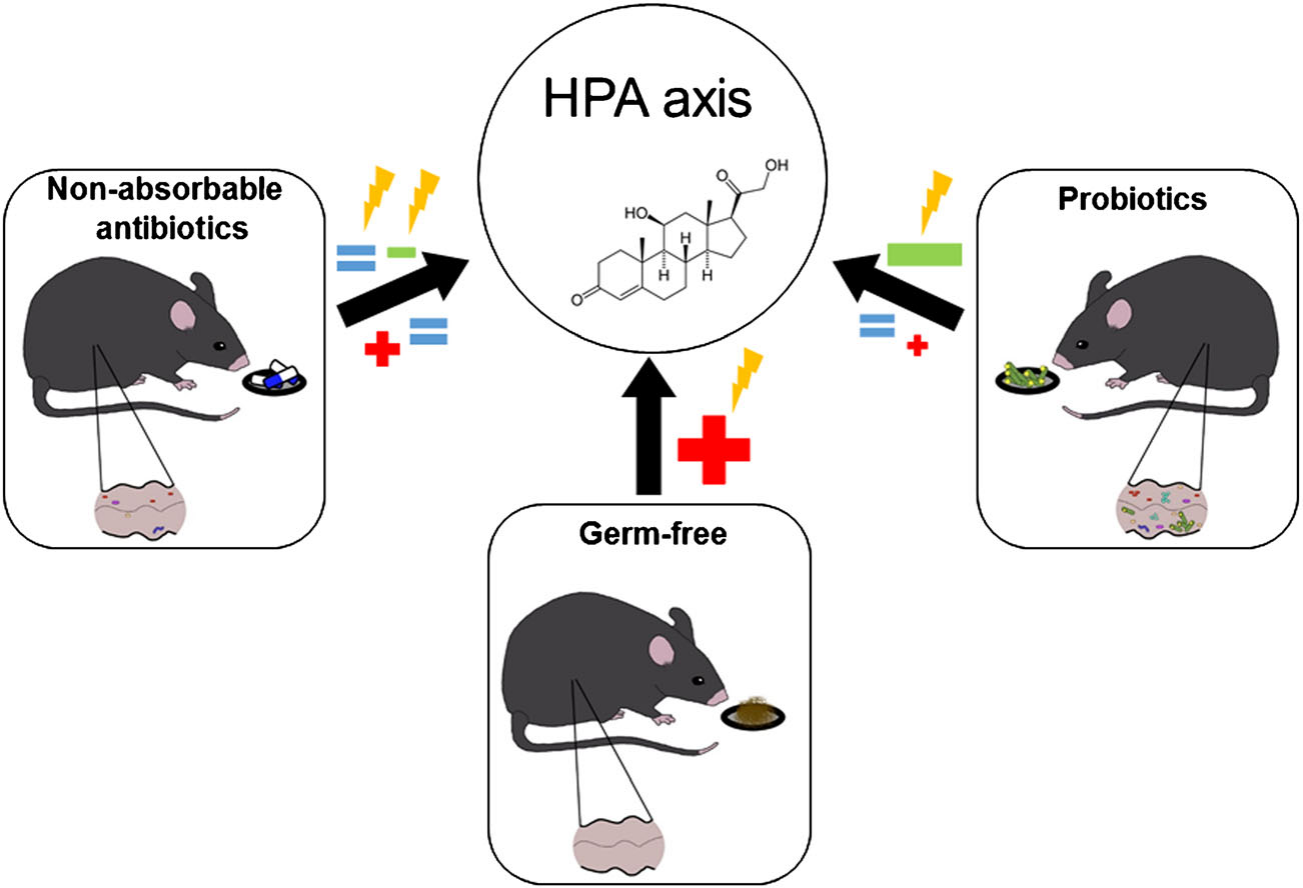
Effects of gut microbiota manipulation on the hypothalamus–pituitary–adrenal (HPA) axis. Manipulation of the gut microbiota is able to activate (+) or inhibit (–) the HPA axis under basal conditions or in response to various stressors ()

The studies reviewed here show inconsistent effects of antibiotic-induced dysbiosis on HPA axis activity, making it difficult to propose a comprehensive framework for the interaction between the gut microbiota and the neuroendocrine system (Fig. 3). The inconsistent findings in this respect may be related to a number of confounding factors, including differences in the animal’s species, strain, and sex, the choice of antibiotics, and/or treatment regimens. Narrowing down the effects of antibiotics to particular changes in the intestinal microbiota composition is often not possible because the microbiota composition has not been or only partially determined or because the microbial community at baseline differs between studies. Furthermore, an effect of the antibiotics themselves on the observed parameters cannot be excluded as is also discussed in the next section.

## GF *versus* Antibiotic-Induced Dysbiosis Models

One of the major challenges of current microbiota research is to obtain evidence for a causal involvement of the gut microbiota in particular physiological and pathophysiological processes. The major experimental approaches to this question rest currently on the use of GF rodents, on the one hand, and dysbiotic animals due to antibiotic treatment, on the other hand. Both approaches have advantages and disadvantages and differ in their translational value, given that GF conditions cannot be studied in humans. The antibiotic-induced dysbiosis model offers the opportunity to study microbial changes and its (patho)physiological consequences in animals born and raised with a conventional/normal microbiota. In conducting valid antibiotic-based studies, special attention needs to be paid to the selection of antibiotics and their pharmacokinetics, because the antibiotics themselves may directly affect the processes (e.g., neuroendocrine responses) under study. Hence, it is important to choose antibiotics, that are not or only minimally absorbed from the gastrointestinal tract, and to validate this condition in the experimental animal species used [137]. In this respect, the approach pursued by Bercik et al. [103] represents a benchmark model for the study of the implications of antibiotic-induced dysbiosis in mouse behavior and brain biochemistry. Oral administration of an antibiotic mix to SPF mice was found to cause gut dysbiosis, to increase exploratory behavior, and to alter BDNF levels in the brain. The involvement of the gut microbiota in the antibiotic-induced behavioral disturbances was validated by the finding that gut dysbiosis and behavioral changes were seen only after oral, but not intraperitoneal, administration of antibiotics. In addition, no behavioral changes were observed in GF mice treated with the antibiotic mix by the oral route [103]. Measuring the presence of the antibiotics in blood and the organs under study is a further approach to identify the sites of antibiotic action and to rule out direct effects on organs such as the brain [129]. Studies involving antibiotics that are readily absorbed from the gastrointestinal tract and even are able to penetrate the blood–brain barrier are thus inconclusive with regard to a relationship between gut microbiota, brain function, and behavior. Such an uncertainty, for instance, applies to the antibiotic metronidazole [137, 138], which not only is absorbed from the intestine and enters the brain, but may also exert neurotoxic effects [139, 140].

## Conclusion

Studies involving oral administration of probiotics or nonabsorbable antibiotics, as well as analysis of GF animals, reveal a multidirectional interplay between the gut microbiota and neuroendocrine system. The demonstration of homeostatic effects of probiotics on neuroendocrine physiology has considerable translational value as it hints at new opportunities of therapeutic intervention. In fact, the majority of preclinical data underpin the potential of certain probiotic strains to exert beneficial effects on the gut–brain axis, especially under conditions of stress. The beneficial effects are likely mediated via immunomodulatory effects of the probiotics and their ability to decrease intestinal translocation of microbial constituents across the intestinal barrier. However, despite this encouraging evidence the underlying mechanisms, as well as the clinical efficacy of these probiotic strains, await to be demonstrated. In contrast to the mainly inhibitory effects of probiotics on HPA axis activity, increased activity of the HPA axis is a consistent finding in GF mice, which can be reversed when the gut is colonized within a certain time window early in life. While this observation points toward a central role of the gut microbiota in the development and regulation of the HPA axis, the underlying processes remain elusive, given the many systems that are affected in GF mice, including immune system, intestinal function, metabolism, and brain development. Finally, oral antibiotic-induced manipulation of the gut microbiota has also been demonstrated to affect the neuroendocrine system, although in a somewhat disparate manner. On the one hand, similar to the GF setting, antibiotic-induced disruption of the microbiota has been reported to increase corticosterone levels, whereas, on the other hand, similar to effects of probiotics, protective effects of antibiotic-induced reduction of the gut microbiota under conditions of stress have also been shown. The latter effect could be explained by a decrease of microbiota-related activation of the immune system in response to stress. Thus, oral antibiotics could exert either beneficial or harmful effects on gut microbiota and neuroendocrine system, depending in the pre-existing composition of the gut microbiota, its interaction with the antibiotics, off-target effects of the antibiotic (e.g., microbiota-independent actions on immune and endocrine system as well as CNS) and environmental influences.

Unravelling the precise mechanisms that underlie the multidirectional communication between gut microbiota and neuroendocrine system could lead to new therapeutic possibilities for disorders of the neuroendocrine system through controlled manipulation of the gut microbiota.

## Electronic Supplementary Material

ESM 1 (PDF 1224 kb)

## References

[1] Toni R (2004). The neuroendocrine system: organization and homeostatic role. J Endocrinol Invest..

[2] Prevot V (2010). Plasticity of neuroendocrine systems. Eur J Neurosci..

[3] Smith SM, Vale WW (2006). The role of the hypothalamic-pituitary-adrenal axis in neuroendocrine responses to stress. Dialogues Clin Neurosci..

[4] Keller-Wood ME, Dallman MF (1984). Corticosteroid inhibition of ACTH secretion. Endocr Rev..

[5] Dinan TG, Cryan JF (2012). Regulation of the stress response by the gut microbiota: implications for psychoneuroendocrinology. Psychoneuroendocrinology..

[6] O'Mahony SM, Clarke G, Dinan TG, Cryan JF (2017). Early-life adversity and brain development: Is the microbiome a missing piece of the puzzle?. Neuroscience..

[7] Juruena MF, Bocharova M, Agustini B, Young AH. Atypical depression and non-atypical depression: Is HPA axis function a biomarker? A systematic review. J Affect Disord. 2017 Oct 6 [Epub ahead print]. 10.1016/j.jad.2017.09.052 29150144

[8] Videlock EJ, Shih W, Adeyemo M (2016). The effect of sex and irritable bowel syndrome on HPA axis response and peripheral glucocorticoid receptor expression. Psychoneuroendocrinology..

[9] Whitehead WE, Crowell MD, Robinson JC, Heller BR, Schuster MM (1992). Effects of stressful life events on bowel symptoms: subjects with irritable bowel syndrome compared with subjects without bowel dysfunction. Gut..

[10] Bradford K, Shih W, Videlock EJ (2012). Association between early adverse life events and irritable bowel syndrome. Clin Gastroenterol Hepatol.

[11] Liu B, Liu J, Wang M, Zhang Y, Li L (2017). From serotonin to neuroplasticity: evolvement of theories for major depressive disorder. Front Cell Neurosci..

[12] Heim C, Newport DJ, Heit S (2000). Pituitary-adrenal and autonomic responses to stress in women after sexual and physical abuse in childhood. JAMA..

[13] de Punder K, Pruimboom L (2015). Stress induces endotoxemia and low-grade inflammation by increasing barrier permeability. Front Immunol..

[14] Kelly JR, Kennedy PJ, Cryan JF, Dinan TG, Clarke G, Hyland NP (2015). Breaking down the barriers: the gut microbiome, intestinal permeability and stress-related psychiatric disorders. Front Cell Neurosci..

[15] Camilleri M, Lasch K, Zhou W (2012). Irritable bowel syndrome: methods, mechanisms, and pathophysiology. The confluence of increased permeability, inflammation, and pain in irritable bowel syndrome. Am J Physiol Gastrointest Liver Physiol..

[16] Slyepchenko A, Maes M, Jacka FN (2017). Gut microbiota, bacterial translocation, and interactions with diet: pathophysiological links between major depressive disorder and non-communicable medical comorbidities. Psychother Psychosom..

[17] Rajilic-Stojanovic M, Jonkers DM, Salonen A (2015). Intestinal microbiota and diet in IBS: causes, consequences, or epiphenomena?. Am J Gastroenterol..

[18] Collins SM (2016). The intestinal microbiota in the irritable bowel syndrome. Int Rev Neurobiol..

[19] Kelly JR, Borre Y, O'Brien C, et al. Transferring the blues: Depression-associated gut microbiota induces neurobehavioural changes in the rat. J Psychiatr Res. 2016;82:109-118. 10.1016/j.jpsychires.2016.07.019 27491067

[20] Naseribafrouei A, Hestad K, Avershina E (2014). Correlation between the human fecal microbiota and depression. Neurogastroenterol Motil..

[21] Jiang H, Ling Z, Zhang Y (2015). Altered fecal microbiota composition in patients with major depressive disorder. Brain Behav Immun..

[22] Zheng P, Zeng B, Zhou C (2016). Gut microbiome remodeling induces depressive-like behaviors through a pathway mediated by the host's metabolism. Mol Psychiatry..

[23] De Palma G, Lynch MD, Lu J, et al. Transplantation of fecal microbiota from patients with irritable bowel syndrome alters gut function and behavior in recipient mice. Sci Transl Med. 2017;9(379). 10.1126/scitranslmed.aaf6397 28251905

[24] Doolin K, Farrell C, Tozzi L, Harkin A, Frodl T, O'Keane V. Diurnal hypothalamic-pituitary-adrenal axis measures and inflammatory marker correlates in major depressive disorder. Int J Mol Sci. 2017;18(10). 10.3390/ijms18102226 PMC5666905 29064428

[25] Holzer P, Reichmann F, Farzi A, Neuropeptide Y (2012). peptide YY and pancreatic polypeptide in the gut-brain axis. Neuropeptides..

[26] Clarke G, Stilling RM, Kennedy PJ, Stanton C, Cryan JF, Dinan TG (2014). Minireview: gut microbiota: the neglected endocrine organ. Mol Endocrinol..

[27] El Aidy S, Dinan TG, Cryan JF (2015). Gut microbiota: the conductor in the orchestra of immune-neuroendocrine communication. Clin Ther..

[28] Sherwin E, Sandhu KV, Dinan TG, Cryan JF (2016). May the force be with you: the light and dark sides of the microbiota-gut-brain axis in neuropsychiatry. CNS Drugs..

[29] Meaney MJ, Aitken DH, Bhatnagar S, Sapolsky RM (1991). Postnatal handling attenuates certain neuroendocrine, anatomical, and cognitive dysfunctions associated with aging in female rats. Neurobiol Aging..

[30] Singh-Taylor A, Molet J, Jiang S, et al. NRSF-dependent epigenetic mechanisms contribute to programming of stress-sensitive neurons by neonatal experience, promoting resilience. Mol Psychiatry 2017 Jan 10 [Epub ahead of print]. 10.1038/mp.2016.240 PMC5503824 28070121

[31] van Bodegom M, Homberg JR, Henckens M (2017). Modulation of the hypothalamic-pituitary-adrenal axis by early life stress exposure. Front Cell Neurosci..

[32] Levitt NS, Lindsay RS, Holmes MC, Seckl JR (1996). Dexamethasone in the last week of pregnancy attenuates hippocampal glucocorticoid receptor gene expression and elevates blood pressure in the adult offspring in the rat. Neuroendocrinology..

[33] Sadler TR, Nguyen PT, Yang J (2011). Antenatal maternal stress alters functional brain responses in adult offspring during conditioned fear. Brain Res..

[34] Son GH, Geum D, Chung S (2006). Maternal stress produces learning deficits associated with impairment of NMDA receptor-mediated synaptic plasticity. J Neurosci..

[35] Sowa J, Bobula B, Glombik K, Slusarczyk J, Basta-Kaim A, Hess G (2015). Prenatal stress enhances excitatory synaptic transmission and impairs long-term potentiation in the frontal cortex of adult offspring rats. PLOS ONE..

[36] Herman JP, McKlveen JM, Ghosal S (2016). Regulation of the hypothalamic-pituitary-adrenocortical stress response. Compr Physiol..

[37] Holzer P, Wultsch T, Edelsbrunner M (2007). Increase in gastric acid-induced afferent input to the brainstem in mice with gastritis. Neuroscience..

[38] Dantzer R, O'Connor JC, Freund GG, Johnson RW, Kelley KW (2008). From inflammation to sickness and depression: when the immune system subjugates the brain. Nat Rev Neurosci..

[39] Danese A, Baldwin JR (2017). Hidden Wounds? Inflammatory links between childhood trauma and psychopathology. Annu Rev Psychol..

[40] Shirtcliff EA, Coe CL, Pollak SD (2009). Early childhood stress is associated with elevated antibody levels to herpes simplex virus type 1. Proc Natl Acad Sci U S A..

[41] Weaver IC, Cervoni N, Champagne FA (2004). Epigenetic programming by maternal behavior. Nat Neurosci..

[42] Klengel T, Mehta D, Anacker C (2013). Allele-specific FKBP5 DNA demethylation mediates gene-childhood trauma interactions. Nat Neurosci..

[43] Bailey MT, Coe CL (1999). Maternal separation disrupts the integrity of the intestinal microflora in infant rhesus monkeys. Dev Psychobiol..

[44] Lyte M, Ernst S (1992). Catecholamine induced growth of gram negative bacteria. Life Sci..

[45] Serrats J, Schiltz JC, Garcia-Bueno B, van Rooijen N, Reyes TM, Sawchenko PE (2010). Dual roles for perivascular macrophages in immune-to-brain signaling. Neuron..

[46] Arentsen T, Qian Y, Gkotzis S (2017). The bacterial peptidoglycan-sensing molecule Pglyrp2 modulates brain development and behavior. Mol Psychiatry..

[47] Clarke TB, Davis KM, Lysenko ES, Zhou AY, Yu Y, Weiser JN (2010). Recognition of peptidoglycan from the microbiota by Nod1 enhances systemic innate immunity. Nat Med..

[48] Farzi A, Reichmann F, Meinitzer A (2015). Synergistic effects of NOD1 or NOD2 and TLR4 activation on mouse sickness behavior in relation to immune and brain activity markers. Brain Behav Immun..

[49] Mayerhofer R, Frohlich EE, Reichmann F (2017). Diverse action of lipoteichoic acid and lipopolysaccharide on neuroinflammation, blood-brain barrier disruption, and anxiety in mice. Brain Behav Immun..

[50] Shanks N, Larocque S, Meaney MJ (1995). Neonatal endotoxin exposure alters the development of the hypothalamic-pituitary-adrenal axis: early illness and later responsivity to stress. J Neurosci..

[51] Mouihate A, Galic MA, Ellis SL, Spencer SJ, Tsutsui S, Pittman QJ (2010). Early life activation of toll-like receptor 4 reprograms neural anti-inflammatory pathways. J Neurosci..

[52] Ong LK, Fuller EA, Sominsky L, Hodgson DM, Dunkley PR, Dickson PW (2017). Early life peripheral lipopolysaccharide challenge reprograms catecholaminergic neurons. Sci Rep..

[53] Chang PV, Hao L, Offermanns S, Medzhitov R (2014). The microbial metabolite butyrate regulates intestinal macrophage function via histone deacetylase inhibition. Proc Natl Acad Sci U S A..

[54] Usami M, Kishimoto K, Ohata A (2008). Butyrate and trichostatin A attenuate nuclear factor kappaB activation and tumor necrosis factor alpha secretion and increase prostaglandin E2 secretion in human peripheral blood mononuclear cells. Nutr Res..

[55] Furusawa Y, Obata Y, Fukuda S (2013). Commensal microbe-derived butyrate induces the differentiation of colonic regulatory T cells. Nature..

[56] Rooks MG, Garrett WS (2016). Gut microbiota, metabolites and host immunity. Nat Rev Immunol..

[57] Erny D, Hrabe de Angelis AL, Jaitin D (2015). Host microbiota constantly control maturation and function of microglia in the CNS. Nat Neurosci..

[58] Castillo-Ruiz A, Mosley M, George AJ (2018). The microbiota influences cell death and microglial colonization in the perinatal mouse brain. Brain Behav Immun.

[59] Bilimoria PM, Stevens B (2015). Microglia function during brain development: new insights from animal models. Brain Res..

[60] Schroeder FA, Lin CL, Crusio WE, Akbarian S (2007). Antidepressant-like effects of the histone deacetylase inhibitor, sodium butyrate, in the mouse. Biol Psychiatry..

[61] Han A, Sung YB, Chung SY, Kwon MS (2014). Possible additional antidepressant-like mechanism of sodium butyrate: targeting the hippocampus. Neuropharmacology..

[62] De Silva A, Bloom SR (2012). Gut hormones and appetite control: a focus on PYY and GLP-1 as therapeutic targets in obesity. Gut Liver..

[63] Brooks L, Viardot A, Tsakmaki A (2017). Fermentable carbohydrate stimulates FFAR2-dependent colonic PYY cell expansion to increase satiety. Mol Metab..

[64] Breton J, Tennoune N, Lucas N (2016). Gut commensal *E. coli* proteins activate host satiety pathways following nutrient-induced bacterial growth. Cell Metab..

[65] Tennoune N, Chan P, Breton J (2014). Bacterial ClpB heat-shock protein, an antigen-mimetic of the anorexigenic peptide alpha-MSH, at the origin of eating disorders. Transl Psychiatry..

[66] Duca FA, Swartz TD, Sakar Y, Covasa M (2012). Increased oral detection, but decreased intestinal signaling for fats in mice lacking gut microbiota. PLOS ONE..

[67] Schele E, Grahnemo L, Anesten F, Hallen A, Backhed F, Jansson JO (2013). The gut microbiota reduces leptin sensitivity and the expression of the obesity-suppressing neuropeptides proglucagon (Gcg) and brain-derived neurotrophic factor (Bdnf) in the central nervous system. Endocrinology..

[68] Holzer P, Farzi A (2014). Neuropeptides and the microbiota-gut-brain axis. Adv Exp Med Biol..

[69] Dhakal R, Bajpai VK, Baek KH (2012). Production of gaba (gamma-aminobutyric acid) by microorganisms: a review. Braz J Microbiol.

[70] Asano Y, Hiramoto T, Nishino R (2012). Critical role of gut microbiota in the production of biologically active, free catecholamines in the gut lumen of mice. Am J Physiol Gastrointest Liver Physiol..

[71] Mazzoli R, Pessione E (2016). The neuro-endocrinological role of microbial glutamate and GABA signaling. Front Microbiol..

[72] Moya-Perez A, Perez-Villalba A, Benitez-Paez A, Campillo I, Sanz Y, Bifidobacterium CECT (2017). 7765 modulates early stress-induced immune, neuroendocrine and behavioral alterations in mice. Brain Behav Immun..

[73] Agusti A, Moya-Perez A, Campillo I, et al. *Bifidobacterium pseudocatenulatum* CECT 7765 ameliorates neuroendocrine alterations associated with an exaggerated stress response and anhedonia in obese mice. Mol Neurobiol 2017 Sep 18 [Epub ahead of print]. 10.1007/s12035-017-0768-z 28921462

[74] Desbonnet L, Garrett L, Clarke G, Kiely B, Cryan JF, Dinan TG (2010). Effects of the probiotic *Bifidobacterium infantis* in the maternal separation model of depression. Neuroscience..

[75] Savignac HM, Kiely B, Dinan TG, Cryan JF (2014). Bifidobacteria exert strain-specific effects on stress-related behavior and physiology in BALB/c mice. Neurogastroenterol Motil..

[76] Barouei J, Moussavi M, Hodgson DM (2012). Effect of maternal probiotic intervention on HPA axis, immunity and gut microbiota in a rat model of irritable bowel syndrome. PLOS ONE..

[77] Bravo JA, Forsythe P, Chew MV (2011). Ingestion of *Lactobacillus* strain regulates emotional behavior and central GABA receptor expression in a mouse via the vagus nerve. Proc Natl Acad Sci U S A.

[78] Bharwani A, Mian MF, Surette MG, Bienenstock J, Forsythe P (2017). Oral treatment with *Lactobacillus rhamnosus* attenuates behavioural deficits and immune changes in chronic social stress. BMC Med..

[79] Kelly JR, Allen AP, Temko A (2017). Lost in translation? The potential psychobiotic *Lactobacillus rhamnosus* (JB-1) fails to modulate stress or cognitive performance in healthy male subjects. Brain Behav Immun..

[80] Ait-Belgnaoui A, Durand H, Cartier C (2012). Prevention of gut leakiness by a probiotic treatment leads to attenuated HPA response to an acute psychological stress in rats. Psychoneuroendocrinology..

[81] Ait-Belgnaoui A, Colom A, Braniste V (2014). Probiotic gut effect prevents the chronic psychological stress-induced brain activity abnormality in mice. Neurogastroenterol Motil..

[82] Gareau MG, Jury J, MacQueen G, Sherman PM, Perdue MH (2007). Probiotic treatment of rat pups normalises corticosterone release and ameliorates colonic dysfunction induced by maternal separation. Gut..

[83] Vanhaecke T, Aubert P, Grohard PA, et al. *L. fermentum* CECT 5716 prevents stress-induced intestinal barrier dysfunction in newborn rats. Neurogastroenterol Motil. 2017;29(8). 10.1111/nmo.13069 28370715

[84] Abildgaard A, Elfving B, Hokland M, Wegener G, Lund S (2017). Probiotic treatment reduces depressive-like behaviour in rats independently of diet. Psychoneuroendocrinology..

[85] Rothhammer V, Mascanfroni ID, Bunse L (2016). Type I interferons and microbial metabolites of tryptophan modulate astrocyte activity and central nervous system inflammation via the aryl hydrocarbon receptor. Nat Med..

[86] Andersson H, Tullberg C, Ahrne S (2016). Oral administration of *Lactobacillus plantarum* 299v reduces cortisol levels in human saliva during examination induced stress: a randomized, double-blind controlled trial. Int J Microbiol..

[87] Messaoudi M, Lalonde R, Violle N (2011). Assessment of psychotropic-like properties of a probiotic formulation (*Lactobacillus helveticus* R0052 and *Bifidobacterium longum* R0175) in rats and human subjects. Br J Nutr..

[88] Allen AP, Hutch W, Borre YE (2016). *Bifidobacterium longum* 1714 as a translational psychobiotic: modulation of stress, electrophysiology and neurocognition in healthy volunteers. Transl Psychiatry..

[89] Burokas A, Arboleya S, Moloney RD (2017). Targeting the microbiota-gut-brain axis: prebiotics have anxiolytic and antidepressant-like effects and reverse the impact of chronic stress in mice. Biol Psychiatry..

[90] Schmidt K, Cowen PJ, Harmer CJ, Tzortzis G, Errington S, Burnet PW (2015). Prebiotic intake reduces the waking cortisol response and alters emotional bias in healthy volunteers. Psychopharmacology (Berl)..

[91] Barrera-Bugueno C, Realini O, Escobar-Luna J (2017). Anxiogenic effects of a *Lactobacillus*, inulin and the synbiotic on healthy juvenile rats. Neuroscience..

[92] Lu B, Nagappan G, BDNF LY (2014). synaptic plasticity, cognitive function, and dysfunction. Handb Exp Pharmacol..

[93] Sudo N, Chida Y, Aiba Y (2004). Postnatal microbial colonization programs the hypothalamic-pituitary-adrenal system for stress response in mice. J Physiol..

[94] Duman RS (2014). Pathophysiology of depression and innovative treatments: remodeling glutamatergic synaptic connections. Dialogues Clin Neurosci..

[95] Cohen SM, Tsien RW, Goff DC, Halassa MM (2015). The impact of NMDA receptor hypofunction on GABAergic neurons in the pathophysiology of schizophrenia. Schizophr Res..

[96] Roceri M, Hendriks W, Racagni G, Ellenbroek BA, Riva MA (2002). Early maternal deprivation reduces the expression of BDNF and NMDA receptor subunits in rat hippocampus. Mol Psychiatry..

[97] Diaz Heijtz R, Wang S, Anuar F (2011). Normal gut microbiota modulates brain development and behavior. Proc Natl Acad Sci U S A..

[98] Neufeld KM, Kang N, Bienenstock J, Foster JA (2011). Reduced anxiety-like behavior and central neurochemical change in germ-free mice. Neurogastroenterol Motil..

[99] Clarke G, Grenham S, Scully P (2013). The microbiome-gut-brain axis during early life regulates the hippocampal serotonergic system in a sex-dependent manner. Mol Psychiatry..

[100] De Palma G, Blennerhassett P, Lu J (2015). Microbiota and host determinants of behavioural phenotype in maternally separated mice. Nat Commun..

[101] Nishino R, Mikami K, Takahashi H (2013). Commensal microbiota modulate murine behaviors in a strictly contamination-free environment confirmed by culture-based methods. Neurogastroenterol Motil..

[102] Crumeyrolle-Arias M, Jaglin M, Bruneau A (2014). Absence of the gut microbiota enhances anxiety-like behavior and neuroendocrine response to acute stress in rats. Psychoneuroendocrinology..

[103] Bercik P, Denou E, Collins J (2011). The intestinal microbiota affect central levels of brain-derived neurotropic factor and behavior in mice. Gastroenterology..

[104] Phelps D, Brinkman NE, Keely SP (2017). Microbial colonization is required for normal neurobehavioral development in zebrafish. Sci Rep..

[105] Weiss SM, Wadsworth G, Fletcher A, Dourish CT (1998). Utility of ethological analysis to overcome locomotor confounds in elevated maze models of anxiety. Neurosci Biobehav Rev..

[106] Strekalova T, Spanagel R, Dolgov O, Bartsch D (2005). Stress-induced hyperlocomotion as a confounding factor in anxiety and depression models in mice. Behav Pharmacol..

[107] Round JL, Mazmanian SK (2009). The gut microbiota shapes intestinal immune responses during health and disease. Nat Rev Immunol..

[108] Lee YK, Menezes JS, Umesaki Y, Mazmanian SK (2011). Proinflammatory T-cell responses to gut microbiota promote experimental autoimmune encephalomyelitis. Proc Natl Acad Sci U S A.

[109] HJ W, Ivanov II, Darce J (2010). Gut-residing segmented filamentous bacteria drive autoimmune arthritis via T helper 17 cells. Immunity..

[110] Olszak T, An D, Zeissig S (2012). Microbial exposure during early life has persistent effects on natural killer T cell function. Science..

[111] Smith CJ, Emge JR, Berzins K (2014). Probiotics normalize the gut-brain-microbiota axis in immunodeficient mice. Am J Physiol Gastrointest Liver Physiol..

[112] Braniste V, Al-Asmakh M, Kowal C (2014). The gut microbiota influences blood-brain barrier permeability in mice. Sci Transl Med.

[113] Matsumoto M, Kibe R, Ooga T (2013). Cerebral low-molecular metabolites influenced by intestinal microbiota: a pilot study. Front Syst Neurosci..

[114] Wikoff WR, Anfora AT, Liu J (2009). Metabolomics analysis reveals large effects of gut microflora on mammalian blood metabolites. Proc Natl Acad Sci U S A..

[115] Stilling RM, Ryan FJ, Hoban AE (2015). Microbes & neurodevelopment—absence of microbiota during early life increases activity-related transcriptional pathways in the amygdala. Brain Behav Immun..

[116] Luczynski P, Whelan SO, O'Sullivan C (2016). Adult microbiota-deficient mice have distinct dendritic morphological changes: differential effects in the amygdala and hippocampus. Eur J Neurosci..

[117] Hoban AE, Stilling RM, M Moloney G, et al. Microbial regulation of microRNA expression in the amygdala and prefrontal cortex. Microbiome. 2017;5(1):102. 10.1186/s40168-017-0321-3 PMC5571609 28838324

[118] Nakata K, Sugi Y, Narabayashi H (2017). Commensal microbiota-induced microRNA modulates intestinal epithelial permeability through the small GTPase ARF4. J Biol Chem..

[119] Smith K, McCoy KD, Macpherson AJ (2007). Use of axenic animals in studying the adaptation of mammals to their commensal intestinal microbiota. Semin Immunol..

[120] Chen HF, Su HM (2013). Exposure to a maternal n-3 fatty acid-deficient diet during brain development provokes excessive hypothalamic-pituitary-adrenal axis responses to stress and behavioral indices of depression and anxiety in male rat offspring later in life. J Nutr Biochem..

[121] Sartori SB, Whittle N, Hetzenauer A, Singewald N (2012). Magnesium deficiency induces anxiety and HPA axis dysregulation: modulation by therapeutic drug treatment. Neuropharmacology..

[122] Marissal-Arvy N, Hamiani R, Richard E, Moisan MP, Pallet V, Vitamin A (2013). regulates hypothalamic-pituitary-adrenal axis status in LOU/C rats. J Endocrinol..

[123] Pusceddu MM, El Aidy S, Crispie F (2015). N-3 polyunsaturated fatty acids (PUFAs) reverse the impact of early-life stress on the gut microbiota. PLOS ONE.

[124] Widmaier EP, Rosen K, Abbott B (1992). Free fatty acids activate the hypothalamic-pituitary-adrenocortical axis in rats. Endocrinology..

[125] Lanfranco F, Giordano R, Pellegrino M (2004). Free fatty acids exert an inhibitory effect on adrenocorticotropin and cortisol secretion in humans. J Clin Endocrinol Metab..

[126] Bailey MT, Dowd SE, Parry NM, Galley JD, Schauer DB, Lyte M (2010). Stressor exposure disrupts commensal microbial populations in the intestines and leads to increased colonization by *Citrobacter rodentium*. InfectImmun..

[127] Scheer S, Medina TS, Murison A (2017). Early-life antibiotic treatment enhances the pathogenicity of CD4+ T cells during intestinal inflammation. J Leukoc Biol..

[128] Trobonjaca Z, Leithauser F, Moller P (2001). MHC-II-independent CD4+ T cells induce colitis in immunodeficient RAG-/- hosts. J Immunol..

[129] Fröhlich EE, Farzi A, Mayerhofer R (2016). Cognitive impairment by antibiotic-induced gut dysbiosis: Analysis of gut microbiota-brain communication. Brain Behav Immun..

[130] Farzi A, Reichmann F, Holzer P (2015). The homeostatic role of neuropeptide Y in immune function and its impact on mood and behaviour. Acta Physiol (Oxf).

[131] Wang T, Hu X, Liang S (2015). *Lactobacillus fermentum* NS9 restores the antibiotic induced physiological and psychological abnormalities in rats. Benef Microbes..

[132] O'Mahony SM, Felice VD, Nally K (2014). Disturbance of the gut microbiota in early-life selectively affects visceral pain in adulthood without impacting cognitive or anxiety-related behaviors in male rats. Neuroscience..

[133] Desbonnet L, Clarke G, Traplin A (2015). Gut microbiota depletion from early adolescence in mice: Implications for brain and behaviour. Brain Behav Immun..

[134] Hoban AE, Moloney RD, Golubeva AV (2016). Behavioural and neurochemical consequences of chronic gut microbiota depletion during adulthood in the rat. Neuroscience..

[135] Garate I, Garcia-Bueno B, Madrigal JL (2011). Origin and consequences of brain Toll-like receptor 4 pathway stimulation in an experimental model of depression. J Neuroinflammation.

[136] Aguilera M, Vergara P, Martinez V (2013). Stress and antibiotics alter luminal and wall-adhered microbiota and enhance the local expression of visceral sensory-related systems in mice. Neurogastroenterol Motil..

[137] Kim S, Covington A, Pamer EG (2017). The intestinal microbiota: antibiotics, colonization resistance, and enteric pathogens. Immunol Rev..

[138] Nau R, Sorgel F, Eiffert H (2010). Penetration of drugs through the blood-cerebrospinal fluid/blood-brain barrier for treatment of central nervous system infections. Clin Microbiol Rev..

[139] Roy U, Panwar A, Pandit A, Das SK, Joshi B (2016). Clinical and neuroradiological spectrum of metronidazole induced encephalopathy: our experience and the review of literature. J Clin Diagn Res..

[140] Goolsby TA, Jakeman B, Gaynes RP. Clinical relevance of metronidazole and peripheral neuropathy: a systematic review of the literature. Int J Antimicrob Agents 2017 Sep 5 [Epub ahead of print]. 10.1016/j.ijantimicag.2017.08.033 28887203

